# Effectiveness of Bivalent mRNA Vaccines in Preventing Symptomatic SARS‐CoV‐2 Infection—Increasing Community Access to Testing Program, United States, January–September 2023

**DOI:** 10.1111/irv.70038

**Published:** 2024-11-10

**Authors:** Allison Avrich Ciesla, Josephine Mak, Lauren E. Roper, Katherine E. Fleming‐Dutra, Zachary R. Smith, Ryan E. Wiegand, Amadea Britton, Joseph Miller, Ruth Link‐Gelles

**Affiliations:** ^1^ National Center for Immunization and Respiratory Diseases Centers for Disease Control and Prevention Atlanta Georgia USA; ^2^ Eagle Health Analytics San Antonio Texas USA; ^3^ Office of Readiness and Response Centers for Disease Control and Prevention Atlanta Georgia USA; ^4^ US Public Health Service Commissioned Corps Rockville Maryland USA

**Keywords:** COVID‐19, Bivalent, SARS‐CoV‐2, Symptomatic Infection

## Abstract

**Background:**

On September 2, 2022, bivalent COVID‐19 mRNA vaccines, were recommended to address reduced effectiveness of COVID‐19 monovalent vaccines during SARS‐CoV‐2 Omicron variant predominance.

**Methods:**

Using national pharmacy‐based SARS‐CoV‐2 testing program data from January 15 to September 11, 2023, this test‐negative, case–control design study assessed bivalent COVID‐19 vaccine effectiveness (VE) against symptomatic infection.

**Results:**

VE against symptomatic infection of a bivalent dose between 2 weeks and 1 month after bivalent vaccination ranged from 46% (95% CI: 38%–52%) for those aged ≥ 65 years to 61% (95% CI 41%–75%) for those aged 12–17 years.

**Conclusion:**

Bivalent vaccines protected against symptomatic infection. However, effectiveness waned over time, emphasizing the need to stay up to date with COVID‐19 vaccination.

## 1 Introduction

On September 2, 2022, bivalent COVID‐19 mRNA vaccines, composed of components from the SARS‐CoV‐2 ancestral and Omicron BA.4/BA.5 strains, were recommended by the Advisory Committee on Immunization Practices (ACIP) to address reduced effectiveness of COVID‐19 monovalent vaccines during SARS‐CoV‐2 Omicron variant predominance [1]. Initial recommendations included a single booster dose for persons aged ≥ 12 years (Pfizer‐BioNTech) and ≥ 18 years (Moderna) who had completed at least a primary series of any Food and Drug Administration–authorized or Food and Drug Administration–approved monovalent vaccine ≥ 2 months earlier [1]. Over the next several months, bivalent COVID‐19 mRNA booster doses were recommended for persons aged 6 months and older, and by April 19, 2023, all mRNA COVID‐19 vaccines were changed to the bivalent formulation, and recommendations were updated with most people aged ≥ 5 years recommended to receive a single bivalent vaccine dose to be up to date (Table S1). On September 11, 2023, an updated (2023–2024) COVID‐19 vaccine formulation was authorized and approved to replace bivalent COVID‐19 vaccines [2].

Observational studies have demonstrated effectiveness of the updated bivalent COVID‐19 mRNA vaccines during earlier Omicron subvariant periods (BA.1, BA.2, BA.4, and BA.5) [3, 4, 5, 6, 7], and early estimates of bivalent vaccine effectiveness among those ≥ 18 years against symptomatic SARS‐COV‐2 infection showed moderate protection, but sufficient time had not passed to assess waning beyond a few months [6, 8].

We analyzed data from a national pharmacy‐based SARS‐CoV‐2 testing program, Increasing Community Access to Testing (ICATT) [9], to estimate age group‐specific duration of protection of at least one bivalent mRNA vaccine dose (compared to those without a bivalent mRNA vaccine dose, but with at least one original monovalent COVID‐19 vaccine dose) among persons aged ≥ 5 years during periods of Omicron XBB subvariant predominance [10].

## 2 Methods

ICATT is a CDC program providing access to no‐cost SARS‐CoV‐2 testing at pharmacies nationwide, prioritizing uninsured patients and areas with high social vulnerability [11, 12]. ICATT VE methods have been described previously [13]. Briefly, at test registration, persons registering for testing (or parents/guardians) report the following information: COVID‐19 vaccination history, current COVID‐19‐like illness symptoms, previous positive SARS‐CoV‐2 test results, and underlying medical conditions. ICATT vendors directly report these data along with SARS‐CoV‐2 test collection date, assay type, and results to CDC. Collection of vaccination history varied by testing vendor, and patients reported either the month and year or the exact date of their most recent dose (Supporting Information A). The number of months between a vaccine dose and testing is a whole number calculated as the difference between the month and year of testing and the month and year of receipt of the vaccine dose. For doses received in the same month or the month before SARS‐CoV‐2 testing, an additional question was asked to specify whether the dose was received ≥ 2 weeks before testing; only those doses received ≥ 2 weeks before testing were included [9].

ICATT data were analyzed from SARS‐CoV‐2 nucleic acid amplification tests (NAATs) performed at Color Health, CVS Pharmacy, eTrueNorth, and Walgreens locations during January 15, 2023, to September 11, 2023, among persons aged ≥ 5 years who reported one or more COVID‐19‐like symptoms (Supporting Information B) and receipt of at least one COVID‐19 vaccine. Case patients were persons who received a positive NAAT result; control patients were those who received a negative NAAT result. Patients who received only original monovalent doses were included if they received any combination, up to four doses, of original monovalent mRNA (Moderna or Pfizer‐BioNTech), Janssen (Johnson & Johnson), or Novavax vaccine; recipients of a single original monovalent mRNA dose (*N* = 11,985) or a single original Novavax dose (*N* = 12) were excluded. Tests among persons fulfilling any of following criteria were excluded from analyses: (1) presence of an immunocompromising condition (based on self‐report at test registration [13]); (2) any COVID‐19 vaccine dose received <14 days before the test date; and (3) persons aged <50 years who received >3 monovalent doses, as this age group was not approved for additional doses at this time [14].

Persons reporting an mRNA booster dose (defined as one or more doses of an mRNA vaccine after receiving 2 mRNA, 2 Novavax, or a single Janssen dose) on or after September 2, 2022, were assumed to have received a bivalent dose. Original monovalent mRNA doses were not authorized for use as booster doses beginning on September 2, 2022 [1]. Persons reporting an mRNA dose on or after April 19, 2023, were considered to have received a bivalent dose, as original monovalent mRNA doses were no longer authorized (Table S1).

Tests from persons who reported a positive SARS‐CoV‐2 test result during the preceding 90 days were excluded to avoid analyzing multiple tests for the same illness episode or reinfections within a relatively short time frame.

A test‐negative design was used to estimate relative VE against symptomatic infection of a bivalent dose by comparing the odds of receipt of a bivalent dose with the odds of no doses in the last 12 months (among cases and controls). Tests from persons who reported receiving no COVID‐19 vaccine doses were excluded from the main analysis; results incorporating this group are included in the Supporting Information.

Age‐group‐stratified odds ratios (ORs) were estimated using multivariable logistic regression; adjusted VE was calculated as (1 − OR) × 100%. Models were adjusted for age, gender, race and ethnicity, pharmacy site census tract social vulnerability index, Health and Human Services region of pharmacy site, calendar week of test (modeled as a categorical variable), underlying conditions (presence vs. absence), and pharmacy chain. Time since last vaccine dose (the main exposure of interest) was modeled as a categorical ordinal variable of 2‐month increments (e.g., 0–1 months since last dose, 2–3 months since last dose, etc.), with the reference group including those without a bivalent vaccine whose last dose was at least 12 months prior.

Several sensitivity analyses were considered. Calendar time of test was evaluated as a continuous linear variable, a spline with 4–10 degrees of freedom, categorical week of testing, and categorical month of testing. Categorical week of testing provided the best model fit. A sensitivity analysis including tests from persons reporting no COVID‐19 vaccine doses was evaluated to examine the differences in this group (Table S3, Model S1). Finally, a sensitivity analysis was carried out to model vaccine effectiveness among persons reporting a more concise list of COVID‐19‐like illness symptoms (cough, fever, congestion, recent loss of sense of smell or taste, or sore throat). This analysis required a subset including only one pharmacy chain that asks specific symptoms during test registration (Table S3, Model S2).

Statistical analyses were performed in R (Version 4.1.2 R Foundation for Statistical Computing, Vienna, Austria).

## 3 Results

**FIGURE 1:**
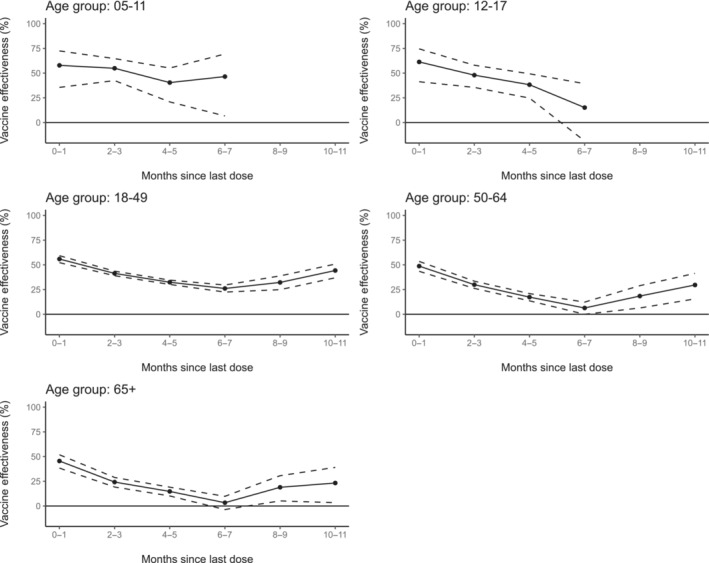
Adjusted vaccine effectiveness of bivalent COVID‐19 doses compared to those without COVID‐19 doses in the preceding 12 months, among those with at least one dose, against symptomatic severe acute respiratory syndrome coronavirus 2 (SARS‐CoV‐2) infection during January 15, 2023, to September 11, 2023, by age‐group and time since most recent bivalent dose receipt. Models adjusted for age, gender, race and ethnicity, pharmacy site census tract social vulnerability index, Health and Human Resources Region of pharmacy site, calendar week of test (modeled as a categorical variable), underlying conditions (presence vs. absence), and pharmacy chain.

A total of 299,400 tests for SARS‐CoV‐2 from 10,678 sites across all states, Puerto Rico and Washington, DC, met inclusion criteria, including 112,333 test‐positive cases and 187,067 test‐negative controls (Table 1). Most persons with eligible tests were 18–49 years old (61%) and female (61%), and 47% identified as non‐Hispanic White. VE against symptomatic infection of a bivalent dose between 2 weeks and 1 month after bivalent vaccination ranged from 46% (95% CI: 38%–52%) for those aged ≥ 65 years to 61% (95% CI 41%–75%) for those aged 12–17 years (Figure 1 and Table S2). VE between the sixth and seventh month after vaccination ranged from 3% (95% CI: −4% to 10%) for those aged ≥ 65 to 46% (95% CI: 7%–69%) for those aged 5–11 years.

**TABLE 1:** Characteristics of persons tested at a pharmacy participation in the Increasing Community Access to Testing program, by SARS‐CoV‐2 infection status and COVID‐19 vaccination status.

Variable	Total	Test Result		COVID‐19 doses	
		*N* (column %)		*N* (row %)	
	*N* (column %)	Positive	Negative	No vaccine in last 12 months	Bivalent
Total (row %)	299,400 (100)	112,333 (38)	187,067 (62)	190,005 (63)	109,395 (37)
Age group					
5–11	3620 (1)	851 (1)	2769 (1)	2109 (58)	1511 (42)
12–17	6488 (2)	2121 (2)	4367 (2)	4835 (75)	1653 (25)
18–49	181,590 (61)	65,051 (58)	116,539 (62)	128,788 (71)	52,802 (29)
50–64	66,560 (22)	27,841 (25)	38,719 (21)	38,650 (58)	27,910 (42)
65+	41,142 (14)	16,469 (15)	24,673 (13)	15,623 (38)	25,519 (62)
Gender					
Female	183,668 (61)	65,836 (59)	117,832 (63)	116,142 (63)	67,526 (37)
Male	113,381 (38)	45,900 (41)	67,481 (36)	72,673 (64)	40,708 (36)
Other	2351 (1)	597 (1)	1754 (1)	1190 (51)	1161 (49)
Race/ethnicity					
Black, non‐Hispanic	37,481 (13)	13,791 (12)	23,690 (13)	27,602 (74)	9879 (26)
Hispanic	59,748 (20)	25,118 (22)	34,630 (19)	46,630 (78)	13,118 (22)
Other NH	43,989 (15)	15,239 (14)	28,750 (15)	26,419 (60)	17,570 (40)
White NH	141,705 (47)	52,217 (46)	89,488 (48)	78,003 (55)	63,702 (45)
Unknown	16,477 (6)	5968 (5)	10,509 (6)	11,351 (69)	5126 (31)
HHS region					
1	16,630 (6)	5489 (5)	11,141 (6)	7715 (46)	8915 (54)
2	25,804 (9)	10,476 (9)	15,328 (8)	16,593 (64)	9211 (36)
3	24,399 (8)	8582 (8)	15,817 (8)	13,164 (54)	11,235 (46)
4	69,037 (23)	25,826 (23)	43,211 (23)	48,439 (70)	20,598 (30)
5	47,322 (16)	18,151 (16)	29,171 (16)	28,533 (60)	18,789 (40)
6	38,505 (13)	14,964 (13)	23,541 (13)	27,240 (71)	11,265 (29)
7	7627 (3)	2714 (2)	4913 (3)	4731 (62)	2896 (38)
8	6158 (2)	2014 (2)	4144 (2)	3854 (63)	2304 (37)
9	56,261 (19)	21,602 (19)	34,659 (19)	35,247 (63)	21,014 (37)
10	7657 (3)	2515 (2)	5142 (3)	4489 (59)	3168 (41)
Social Vulnerability Index					
Mean (std)	0.5 (0.3)	0.5 (0.3)	0.5 (0.3)	0.5 (0.3)	0.5 (0.3)
Reported history of SARS‐CoV‐2 positive test^a^					
None	166,582 (56)	74,975 (67)	91,607 (49)	97,632 (59)	68,950 (41)
Positive > 90 days before current test	132,818 (44)	37,358 (33)	95,460 (51)	92,373 (70)	40,445 (30)
Test type					
Laboratory‐based	203,921 (68)	78,015 (69)	125,906 (67)	121,664 (60)	82,257 (40)
Rapid/point of care	95,479 (32)	34,318 (31)	61,161 (33)	68,341 (72)	27,138 (28)
Self‐reported ≥ 1 chronic underlying condition^b^	86,645 (29)	33,145 (30)	53,500 (29)	49,723 (57)	36,922 (43)
Number of COVID‐19 vaccine doses received					
1	8402 (3)	3370 (3)	5032 (3)	8346 (99)	56 (1)
2	106,526 (36)	41,195 (37)	65,331 (35)	105,789 (99)	737 (1)
3	97,390 (33)	38,240 (34)	59,150 (32)	72,876 (75)	24,514 (25)
4	64,881 (22)	21,020 (19)	43,861 (23)	2723 (4)	62,157 (96)
5	22,043 (7)	8454 (8)	13,589 (7))	270 (1)	21,773 (99)
6	158 (0.05)	54 (0.05)	104 (0.06)	0 (0)	158 (100)
COVID‐19 doses					
No vaccine in last 12 months	190,005 (63)	76,188 (68)	113,817 (61)	190,005 (100)	0 (0)
Bivalent	109,395 (37)	36,145 (32)	73,250 (39)	0 (0)	109,395 (100)

^a^ At test registration, self‐reported history of prior infection is collected. Those indicating prior infection <90 days prior to the test date are excluded from this analysis.

^b^ Underlying risk conditions included on the test registration questionnaire were heart conditions, high blood pressure, overweight or obesity, diabetes, current or former smoker, kidney failure or end stage renal disease, cirrhosis of the liver, and chronic lung disease (e.g., chronic obstructive pulmonary disease, moderate to severe asthma, cystic fibrosis, or pulmonary embolism). This is represented as presence or absence of at least one risk condition.

Variability between unadjusted and adjusted VE estimates (Table S2) was assessed and determined to be largely affected by calendar time, as expected due to changes in both variant and test patterns within the SARS‐CoV‐2 testing program. When including tests from persons reporting no COVID‐19 vaccine doses (Table S3, Model S1), VE estimates were lower for all models (across each age group and time since last dose). When restricting tests to those who reported symptoms within a more concise list of COVID‐19‐like illness symptoms (Table S3, Model S2), VE estimates were largely similar for estimates among age groups ≥ 18 years, whereas more fluctuation was observed at younger age groups (where sample size was greatly reduced).

## 4 Discussion

In this analysis of national pharmacy testing data, bivalent doses of COVID‐19 vaccine provided protection against symptomatic SARS‐CoV‐2 infection among persons ≥ 5 years old during January 15, 2023, to September 11, 2023 (a period of XBB predominance), though protection decreased with more time since vaccination. Vaccination provided protection in all age groups but was lower in adults, especially those aged 65 years and older. Relative VE estimates presented in this analysis should be interpreted in the context of relatively high population immunity to SARS‐CoV‐2, from both prior infection and prior vaccination, and reflects the incremental benefit of bivalent vaccine above that existing immunity.

Among adults aged ≥ 18 years, VE waned through 6–7 months since last dose similar to previous studies of bivalent COVID‐19 vaccines [3, 4, 5, 6, 7, 8]. Increasing VE estimates observed in the adult population after 6–7 months since vaccination may represent residual confounding due to underreported recent SARS‐CoV‐2 infection. This study differs from previous original monovalent booster analyses within this platform where monovalent VE was higher than the observed VE in this study but waned more rapidly [13]. The differential waning between studies provides some evidence that the bivalent COVID‐19 vaccines may have generated a broader immune response to improve protection against Omicron variants circulating during the study period better than the original vaccine formulations after the emergence of the Omicron variant.

There are several important limitations of this study. First, the population included is based on persons accessing pharmacies for SARS‐CoV‐2 testing. Differences in testing practices (including the use of home tests) by presence of symptoms or vaccination status could introduce selection bias. Second, vaccination status and prior infection were self‐reported and subject to recall bias; therefore, there is a potential for misclassification. To lessen the potential for recall bias in relation to vaccination status, individuals were grouped as having a recent dose (bivalent) and no recent dose. As of December 2022, 77.5% [15] of the US population had infection‐induced seroprevalence; however, in this analysis, only 39% of the initial sample reported any prior infection, suggesting underreporting. To reduce potential bias introduced from underreporting of prior infection, we included all individuals unless they reported a recent infection; tests from persons who reported a positive SARS‐CoV‐2 test result during the preceding 90 days were excluded to avoid analyzing multiple tests from the same illness episode or reinfections within a relatively short time frame. Third, the screening survey at test registration did not include information on community or household‐level exposures, individual prevention behaviors (e.g., mask use), or other potential confounders. Those who remain unvaccinated may be meaningfully different from vaccinated individuals. To reduce this potential bias, we present vaccine effectiveness comparing those with a bivalent dose to those without doses in the last 12 months, among individuals reporting at least one dose. Fourth, these data are from pharmacies in regions of higher social vulnerability to better reach underserved communities; therefore, the population included may not be representative of the general US population, or even geographic areas where reporting pharmacy sites are located.

The results from this study emphasize the need to stay up to date on recommended COVID‐19 vaccination.

## Author Contributions

**Allison Avrich Ciesla:** writing – original draft, formal analysis, data curation, methodology, conceptualization, visualization. **Josephine Mak:** writing – review and editing, formal analysis. **Lauren E. Roper:** writing – review and editing, formal analysis. **Katherine E. Fleming‐Dutra:** supervision, conceptualization, data curation, methodology, writing – review and editing. **Zachary R. Smith:** data curation, writing – review and editing, methodology. **Ryan E. Wiegand:** writing – review and editing, conceptualization, methodology. **Amadea Britton:** writing – review and editing, methodology, conceptualization. **Joseph Miller:** funding acquisition, data curation, project administration, supervision, writing – review and editing. **Ruth Link‐Gelles:** supervision, methodology, conceptualization, data curation, writing–review and editing, writing – original draft.

## Ethics Statement

This activity was reviewed by CDC, deemed not research, and was conducted consistent with applicable federal law and CDC policy (see e.g., 45 C.F.R. part 46; 21 C.F.R. part 56; 42 U.S.C. §241[d], 5 U.S.C. §552a, 44 U.S.C. §3501 et seq.).

## Conflicts of Interest

The authors declare no conflicts of interest.

## Supporting information


**Table S1.** Bivalent COVID‐19 mRNA vaccine recommendation datesa.
**Table S2.** Unadjusted and adjusteda vaccine effectiveness of bivalent COVID‐19 doses compared to those without COVID‐19 doses in the preceding 12‐months against symptomatic severe acute respiratory syndrome coronavirus 2 (SARS‐CoV‐2) infection during January 15, 2023‐September 11, 2023, by age group and time since most recent bivalent dose receipt.
**Table S3.**Sensitivity analyses: adjusted vaccine effectiveness of bivalent COVID‐19 doses compared to those without COVID‐19 doses in the preceding 12‐months against symptomatic severe acute respiratory syndrome coronavirus 2 (SARS‐CoV‐2) infection during January 15, 2023‐September 11, 2023, by age group and time since most recent bivalent dose receipt.

## Data Availability

Research data are not shared.
