# Supplementary material for: Effectiveness of Bivalent mRNA Vaccines in Preventing Symptomatic SARS‐CoV‐2 Infection—Increasing Community Access to Testing Program, United States, January–September 2023
Source: Influenza Other Respir Viruses. 2024 Nov 10;18(11):e70038. doi: 10.1111/irv.70038 (PMC11550894; doi:10.1111/irv.70038)
Supplement: Supplementary file 1 — Table S1. Bivalent COVID‐19 mRNA vaccine recommendation datesa. Table S2. Unadjusted and adjusteda vaccine effectiveness of bivalent COVID‐19 doses compared to those without COVID‐19 doses in the preceding 12‐months against symptomatic severe acute respiratory syndrome coronavirus 2 (SARS‐CoV‐2) infection during January 15, 2023‐September 11, 2023, by age group and time since most recent bivalent dose receipt. Table S3.Sensitivity analyses: adjusted vaccine effectiveness of bivalent COVID‐19 doses compared to those without COVID‐19 doses in the preceding 12‐months against symptomatic severe acute respiratory syndrome coronavirus 2 (SARS‐CoV‐2) infection during January 15, 2023‐September 11, 2023, by age group and time since most recent bivalent dose receipt. [file IRV-18-e70038-s001.docx]

Supplement A

At Walgreens, comprising 49% of tests meeting inclusion criteria, test registrants who reported receiving COVID-19 vaccines were asked to report the total number of doses received, the manufacturer of each dose, and the month and year of each dose. At Color Health, eTrueNorth, and CVS Pharmacy, comprising 51% of tests meeting inclusion criteria, test registrants who reported receiving COVID-19 vaccines were asked to report the total number of doses received, the manufacturer of the most recent dose received, and the date of the most recent dose received.

Supplement B

At time of test registration, patients identified COVID-19-like-illness by selecting any of the following: fever, cough, shortness of breath, recent loss of sense of smell or taste, muscle pain, fatigue, chill, headache, sore throat, congestion or runny nose, vomiting, or diarrhea; reported as asymptomatic or symptomatic with ≥1 symptom. Patients indicating any COVID-19-like-illness were included in the analysis.

| **Supplemental Table 1. Bivalent COVID-19 mRNA vaccine recommendation dates^a^** | | | |
| --- | --- | --- | --- |
| **Product** | **Age group** | **Recommended date for booster doses^b^** | **Recommended date regardless of previous vaccine history^c^** |
| Pfizer | 6mo-4y | 12/9/2022^d^ & 3/16/2023^e^ | 4/19/2023 |
|  | 5-11y | 10/12/2022 | 4/19/2023 |
|  | 12-64y | 9/2/2022 | 4/19/2023 |
|  | ≥65y | 9/2/2022 & 4/19/2023^d^ | 4/19/2023 |
| Moderna | 6mo-5y | 12/9/2022^f^ | 4/19/2022 |
|  | 6-17y | 10/12/2022 | 4/19/2023 |
|  | 18-64y | 9/2/2022 | 4/19/2023 |
|  | ≥65y | 9/2/2022 & 4/19/2023^g^ | 4/19/2023 |
| 1. <https://www.cdc.gov/vaccines/covid-19/clinical-considerations/interim-considerations-us-appendix.html> 2. ACIP dates of recommendations for bivalent booster doses. 3. ACIP dates of recommendations for bivalent mRNA doses, regardless of prior vaccination history. From 4/19/2023 to 9/11/2023, all doses, including primary series were bivalent doses. Original monovalent COVID-19 vaccines were no longer authorized. On 9/11/2023 bivalent doses were replaced with updated (2023-2024 Formula) monovalent COVID-19 doses. 4. Children 6 months to 4 years of age who began a Pfizer primary series consisting of only original monovalent doses, were recommended to receive a bivalent dose as part of their 3-dose Pfizer primary series, if they had not completed their Pfizer primary series by 12/9/2022. 5. Children 6 months to 4 years of age who completed a Pfizer primary series with only original monovalent doses, were recommended to receive a bivalent booster dose beginning 3/16/2023 6. Children 6 months to 5 years of age who had a completed Moderna primary series were recommended to receive a bivalent booster dose beginning 12/9/2022. 7. Recommendations were updated that adults aged 65 years and older may receive an additional bivalent booster dose on or after 4/19/2023, even if they had already received a bivalent booster after 9/1/2022. | | | |

Supplement C

| Supplemental Table 2. Unadjusted and adjusted^a^ vaccine effectiveness of bivalent COVID-19 doses compared to those without COVID-19 doses in the preceding 12 months against symptomatic severe acute respiratory syndrome coronavirus 2 (SARS-CoV-2) infection during January 15, 2023-September 11, 2023, by age group and time since most recent bivalent dose receipt | | | |
| --- | --- | --- | --- |
|  |  | | |
| Age Group | Months since last dose | Unadjusted Vaccine Effectiveness (95% Confidence Interval) | Adjusted Vaccine Effectiveness (95% Confidence Interval) |
| 5-11 | 0-1 | 35 (3-56) | 58 (36-72) |
|  | 2-3 | 37 (22-49) | 55 (42-65) |
|  | 4-5 | 35 (16-49) | 40 (21-55) |
|  | 6-7 | 37 (-3-61) | 46 (7-69) |
| 12-17 | 0-1 | 40 (10-60) | 61 (41-75) |
|  | 2-3 | 24 (8-38) | 48 (36-58) |
|  | 4-5 | 23 (8-36) | 38 (25-49) |
|  | 6-7 | 20 (-9-42) | 15 (-19-39) |
| 18-49 | 0-1 | 42 (37-46) | 56 (52-59) |
|  | 2-3 | 23 (20-26) | 41 (39-44) |
|  | 4-5 | 17 (15-20) | 32 (30-35) |
|  | 6-7 | 28 (25-32) | 26 (22-30) |
|  | 8-9 | 18 (10-26) | 32 (25-39) |
|  | 10-11 | 0 (-12-11) | 44 (37-51) |
| 50-64 | 0-1 | 35 (29-41) | 49 (43-53) |
|  | 2-3 | 11 (7-15) | 30 (26-33) |
|  | 4-5 | 4 (-1-7) | 17 (14-21) |
|  | 6-7 | 13 (7-18) | 6 (0-12) |
|  | 8-9 | 5 (-7-17) | 18 (6-29) |
|  | 10-11 | -12 (-33-5) | 30 (16-41) |
| 65+ | 0-1 | 31 (23-39) | 46 (38-52) |
|  | 2-3 | 4 (-1-10) | 24 (19-29) |
|  | 4-5 | -2 (-7-3) | 15 (10-19) |
|  | 6-7 | 10 (4-15) | 3 (-4-10) |
|  | 8-9 | 12 (-1-23) | 19 (5-31) |
|  | 10-11 | -24 (-52--1) | 23 (3-39) |
| a. Models adjusted for age, gender, race and ethnicity, pharmacy site census tract social vulnerability index, Health and Human Resources Region of pharmacy site, calendar week of test (modeled as categorical variable), underlying conditions (presence vs. absence), and pharmacy chain. | | | |

| Supplemental Table 3. Sensitivity analyses: adjusted vaccine effectiveness of bivalent COVID-19 doses compared to those without COVID-19 doses in the preceding 12 months against symptomatic severe acute respiratory syndrome coronavirus 2 (SARS-CoV-2) infection during January 15, 2023-September 11, 2023, by age group and time since most recent bivalent dose receipt | | | | |
| --- | --- | --- | --- | --- |
|  | | *VE% (95%CI)* | | |
| Age Group | Months since last dose* | Original Estimate | Model S1 | Model S2 |
| 5-11 | 0-1 | 58 (36-72) | 41 (12-61) | 40 (-19-70) |
|  | 2-3 | 55 (42-65) | 38 (22-50) | 52 (25-69) |
|  | 4-5 | 40 (21-55) | 13 (-12-33) | 31 (-9-57) |
|  | 6-7 | 46 (7-69) | 16 (-41-50) | 51 (-11-79) |
| 12-17 | 0-1 | 61 (41-75) | 48 (22-66) | 71 (39-86) |
|  | 2-3 | 48 (36-58) | 32 (16-44) | 43 (20-60) |
|  | 4-5 | 38 (25-49) | 17 (-1-31) | 45 (23-61) |
|  | 6-7 | 15 (-19-39) | -24 (-72-10) | 24 (-34-57) |
| 18-49 | 0-1 | 56 (52-59) | 48 (43-52) | 54 (48-60) |
|  | 2-3 | 41 (39-44) | 30 (27-33) | 42 (38-46) |
|  | 4-5 | 32 (30-35) | 19 (16-22) | 33 (29-36) |
|  | 6-7 | 26 (22-30) | 9 (5-13) | 23 (17-29) |
|  | 8-9 | 32 (25-39) | 17 (9-25) | 30 (21-37) |
|  | 10-11 | 44 (37-51) | 37 (29-45) | 43 (35-50) |
| 50-64 | 0-1 | 49 (43-53) | 41 (35-46) | 47 (38-55) |
|  | 2-3 | 30 (26-33) | 19 (15-23) | 31 (24-36) |
|  | 4-5 | 17 (14-21) | 4 (0-8) | 15 (9-21) |
|  | 6-7 | 6 (0-12) | -10 (-17--3) | 3 (-7-12) |
|  | 8-9 | 18 (6-29) | 3 (-11-15) | 17 (2-29) |
|  | 10-11 | 30 (16-41) | 20 (4-33) | 27 (12-40) |
| 65+ | 0-1 | 46 (38-52) | 37 (29-45) | 51 (40-59) |
|  | 2-3 | 24 (19-29) | 13 (8-18) | 24 (17-31) |
|  | 4-5 | 15 (10-19) | 2 (-3-7) | 14 (7-21) |
|  | 6-7 | 3 (-4-10) | -10 (-18--3) | 3 (-7-13) |
|  | 8-9 | 19 (5-31) | 4 (-12-17) | 21 (4-34) |
|  | 10-11 | 23 (3-39) | 10 (-12-28) | 24 (3-40) |
| **Original Model**: **Subset to those with at least 1 COVID-19 vaccine dose.** COVID-19 test result = Month since last dose + age + gender + race/ethnicty + Social Vulnerability Index (high vs low) + HHS Region + Date of testing (as a categorical start of week) + Risk condition (any vs none) + Site Contractor  **Model S1**: **Including those with no reported doses of COVID-19 vaccine.** COVID-19 test result = Month since last dose + age + gender + race/ethnicty + Social Vulnerability Index (high vs low) + HHS Region + Date of testing (as a categorical start of week) + Risk condition (any vs none) + Site Contractor  **Model S2**: **Subset to those indicating specific symptoms (cough, fever, congestion, recent loss of sense of smell or taste, or sore throat) which were only on surveys from Walgreens, so Site Contractor was not included and tests from persons indicating at least 1 COVID-19 vaccine dose.** COVID-19 test result = Month since last dose + age + gender + race/ethnicty + Social Vulnerability Index (high vs low) + HHS Region + Date of testing (as a categorical start of week) + Risk condition (any vs none) | | | | |
